# Family physicians' involvement and self-reported comfort and skill in care of children with behavioral and emotional problems: a population-based survey

**DOI:** 10.1186/1471-2296-6-12

**Published:** 2005-03-11

**Authors:** Anton R Miller, Charlotte Johnston, Anne F Klassen, Stuart Fine, Michael Papsdorf

**Affiliations:** 1 Department of Pediatrics, University of British Columbia, Vancouver BC, Canada; 2 Centre for Community Child Health Research, BC Research Institute for Children's and Women's Health, Children's and Women's Health Centre of BC, Vancouver BC, Canada; 3 Department of Psychology, University of British Columbia, Vancouver BC, Canada; 4 Department of Psychiatry, University of British Columbia, Vancouver BC, Canada

## Abstract

**Background:**

Little is known about general and family practitioners' (GP/FPs') involvement and confidence in dealing with children with common psychosocial problems and mental health conditions. The aims of this study were to ascertain GP/FPs' preferred level of involvement with, and perceived comfort and skill in dealing with children with behavioral problems, social-emotional difficulties, attention-deficit/hyperactivity disorder (ADHD), and mood disorders; and to identify factors associated with GP/FPs' involvement, comfort and skill.

**Methods:**

Postal survey of a representative sample of 801 GP/FPs in British Columbia, Canada, which enquired about level of involvement (from primarily refer out to deal with case oneself); ratings of comfort/skill with assessment/diagnosis and management; beliefs regarding psychosocial problems in children; basic demographics; and practice information.

**Results:**

Surveys were completed by 405 of 629 eligible GP/FPs (64.4%). Over 80% of respondents reported collaborative arrangements with specialists across problem and condition types, although for children with behavior problems or ADHD, more physicians primarily refer (χ^2 ^(1) = 9.0; P < 0.005; and χ^2 ^(1) = 103.9; P < 0.001, respectively). Comfort/skill levels (mean ± s.d) were higher for mood disorders (4.4 ± 1.3) than behavior problems (3.6 ± 1.1; F [3, 1155] = 84.0, P < .0001; effect size = 0.67), but not different from social-emotional difficulties (3.8 ± 1.1) or ADHD (3.9 ± 1.3). Taking primary responsibility for a case was consistently related to self-reported comfort/skill with each condition type (34% to 61% of variance across condition types), while comfort/skill ratings for each condition were related to exposure to relevant continuing medical education (all P ≤ 0.001), and beliefs that these problems are significant and that GP/FPs have a role to play in dealing with them (P values ranged from 0.01 to <0.001).

**Conclusion:**

Supporting GP/FPs in their care for children with common psychosocial and mental health problems should include efforts to bolster their confidence and modify attitudes in relation towards these problems, especially behavior problems and ADHD, possibly within innovative continuing education programs.

## Background

Clinically significant psychosocial and mental health problems affect up to 20% of children and youth presenting for primary care services[1] (we will refer to children and youth as 'children' for purposes of brevity). Primary care physicians, chiefly pediatricians in the United States and parts of Canada, and general and family practitioners (GP/FPs) in other parts of the world, are increasingly expected to participate in, if not assume responsibility for, the care of these children[2,3]. While GP/FPs feel confident in managing common mental health concerns of adults[4], much less is known about their role or about their level of confidence in working with mental health concerns of children, such as depression, attention-deficit/hyperactivity disorder (ADHD), and behavioral and emotional problems[5]. Such information would aid the design of effective health care services for these children, and relevant educational programming for GP/FPs.

The literature relevant to these questions is limited and difficult to interpret. US studies tend to group GP/FPs and pediatricians together, while studies specific to GP/FPs, from European countries and Australia, often focus on approaches to a single condition. The latter group of studies show that GP/FPs frequently lack confidence in relation to ADHD[6], are reluctant to diagnose and manage cases[7,8], and are doubtful that it can be managed solely within primary care.[9] These GP/FPs express a higher level of confidence in care of children with emotional problems, depression and conduct disorders.[6,9] In US studies, primary care physicians are generally not comfortable with the treatment of children with depression, [5,10], and are more likely to refer children with emotional problems than with ADHD to specialized mental health services[5]. Interestingly, GP/FPs tend to be more comfortable than pediatricians in working with children with depression, and more likely to treat such cases themselves[11].

Gender and self-confidence are among the factors that influence GP/FP practice with mental health issues. Female physicians report care of children's psychosocial problems to be less burdensome than do male physicians[12], and, in the management of depression among children and youth, female pediatricians are somewhat more likely to provide counselling and involve other family members [10]. Physician confidence is associated with making fewer referrals to specialists, at least for adult depression [4]. However, little research has examined the factors that affect whether GP/FPs take primary responsibility for behavioral and emotional concerns in children, rather than referring for specialist care, or factors that affect the GP/FPs' self-efficacy in dealing with these children.

To address these issues, we surveyed a representative sample of GP/FPs in the province of British Columbia (BC), Canada. Our aims were to ascertain the preferred level of involvement of GP/FPs in a range of common psychosocial and mental health problems among children, their perceived comfort and competence in dealing with these problems, and the factors associated with differences in levels of involvement and comfort and skill. We enquired about GP/FPs' involvement with conditions that would be included in both formal diagnostic nomenclature (ADHD and mood disorders), and the more loosely-defined set of conditions described collectively as behavior problems and social-emotional difficulties. The latter terms have been commonly used in large scale surveys of primary care practitioners [5], and include a range of psychosocial problems that are mild or 'subthreshold' for formal psychiatric diagnosis [5].

To understand the health services context of this study, GP/FPs provide for most of children's primary care needs in BC, with pediatricians and psychiatrists providing mainly consultative services. The costs of 'medically necessary services' for residents of the province are covered under a universal health insurance coverage plan known across Canada as Medicare, although BC residents who earn above a certain level must contribute to annual premiums. Most physicians are paid on a fee-for-service basis through the provincial Medical Services Plan, based on specialty and service-specific fee schedules. Patients have access to any medical practitioner licensed to practice in BC, but specialists require a referral from a family practitioner to obtain specialist rates of pay. There are also mental health clinics throughout the province, most of which require a referral from medical practitioners or other health care professionals. Access to specialized mental health services is significantly limited by a shortage of these services, especially in rural areas, where pediatrician consultation may the only medical resource available to GP/FPs for child mental health problems. Access to psychologists' services is also limited because these are not covered by Medicare, and not all residents carry insurance for these services. GP/FPs are only remunerated for a limited number of appointments per patient for counselling, which may affect their interest in ongoing treatment.

## Methods

### Study design

Mail out survey of GP/FPs practicing in British Columbia, Canada.

### Participants

In order to obtain a final respondent sample of 10% of eligible GP/FPs in the province, we randomly selected 801 names from a total of 3,953 in a registry provided by the College of Physicians and Surgeons of BC after stratification by Health Region (n = 20) to ensure a representative geographic sample. To participate in this study, physicians had to be in active general or family practice, and to see at least five children per month. From the College registry, we obtained each GP/FP's name, office address and phone number; medical specialty status, including certification in Family Medicine from the College of Family Physicians of Canada; date of birth; date of graduation, and university of graduation. Ethical approval for this study was obtained from the University of British Columbia Behavioral Research Ethics Board.

### Survey instrument

The authors developed a 22-item survey questionnaire, through consultation and consensus, to cover five areas. In relation to children with four clinical conditions, namely behavior problems, social-emotional difficulties, ADHD, and mood disorders, we enquired about (1) number of patients presenting in a typical month; (2) the GP/FP's preferred approach to involvement; and (3) his/her comfort/skill and effectiveness in dealing with each condition. In addition, we assessed (4) general beliefs about children with psychosocial problems; and obtained (5) information about demographic and practice characteristics. Because our expressed aim in this survey was "to learn about how primary care physicians in BC experience providing care to children and youth with behavioral and emotional problems", we did not enquire as to details of the physicians' diagnostic or treatment methods.

Behavior problems were defined as including "disruptive, non-compliant, aggressive, antisocial, oppositional and overactive behaviors"; and social-emotional difficulties as including "low self-esteem, social withdrawal and fearfulness"; and mood disorders as including "anxiety and depression". Our intention in using these terms was to allow the kind of latitude and subjectivity that physicians are accustomed to in their use of these terms in daily practice. We note, however, that the examples given in the Questionnaire to operationalize these terms, would point respondents to think of 'behavior problems' as suggestive of a possible disturbance in the range of disruptive, oppositional and antisocial disorders; and of 'social-emotional difficulties' as possibly suggestive of anxiety or depression. A draft version of the questionnaire was pilot tested with eight GP/FPs who commented on the measure's ease of use, clarity, completeness and relevance. These comments were incorporated into the final version.

*Preferred approach to case involvement *was ascertained by enquiring about the GP/FP's usual pattern of practice regarding children with each clinical condition. Options ranged from "evaluate and manage the problem yourself" to "refer out for evaluation and management", with intermediate arrangements for combining care with specialists.

*Self-reported levels of comfort, skill and effectiveness*GP/FPs were asked to rate their confidence and skill across four domains of clinical activity (comfort with diagnosis/evaluation; comfort with management; skill in diagnosis/evaluation; and effectiveness in management) for each clinical condition. Ratings were made on 7-point scales for each item, anchored by Very Uncomfortable/Unskilled/Ineffective (score of 1) and Very Comfortable/Skilled/Effective (score of 7).

*Beliefs about mental health problems in children *were measured by GP/FPs' degree of agreement with four statements relating to the nature, etiology, evaluation and management of such problems (two of them adapted from the Management of Childhood Depression in Primary Care questionnaire [11]), also using 7-point scales, anchored by Strongly Disagree (score of 1) and Strongly Agree (score of 7).

*Physician personal, demographic and practice characteristics *included gender; practice type (Solo/Group/Walk in/other); practice location (Urban/Rural/Other); hours per week spent in patient care; and participation in continuing medical education activities (CME) covering children with behavioral and emotional problems over the past 5 years; as well as the number of children per month presenting with each condition type, or in whom the GP/FP would consider one of these diagnoses.

### Procedures

Survey packages, including questionnaire, covering letter, prepaid return envelope, and a personalized $15 cheque (that recipients could keep or return as a donation to the hospital's charitable foundation), were mailed out in waves of approximately 200 each between November 2001 and March 2002. The initial mailout was followed 2 weeks later by a thank you/reminder letter, and 6 weeks later by a duplicate survey package to non-respondents. Two weeks later, we phoned the offices of non-responding GP/FPs to encourage participation, elucidate reasons for non-participation, and to try to confirm the GP/FP's eligibility status.

### Data reduction and analysis

In an initial data reduction step, the four comfort/skill and effectiveness items for each condition were subjected to principal components analyses (PCA). These analyses resulted in one component solutions for each condition type that accounted for a large percentage of the total variation in item scores, ranging from 76% to 83%. Each component reflected "dealing with" a particular condition type. For example, the four items concerning comfort and skill in assessing/diagnosing and managing, and effectiveness and skill in managing behavior problems, loaded uniformly highly onto a derived component for "Comfort/skill in dealing with behavior problems". Component scores were therefore created by averaging the four items for each condition type, yielding four scores: Comfort/Skill with behavior problems, social-emotional difficulties, ADHD, and mood disorders, respectively.

To compare Comfort/Skill scores across condition types, we used repeated measures ANOVAs. In all analyses, we adopted a more stringent alpha level of 0.01 to indicate statistical significance (to protect against Type I error rate inflation) and an effect size of 0.5 (Cohen's d) to indicate clinical significance. To discern trends in GP/FP comfort and skill across condition types more clearly, we also characterized respondents' reported scores of 1, 2 or 3 on the relevant items as having "Low" self-reported Comfort/Skill, and those reporting scores of 5, 6 or 7 as having "High" self-reported Comfort/Skill.

Multiple linear regression analyses were used to explore factors associated with Comfort/Skill scores (dependent variable) for each of the condition types. Independent variables included GP/FP background and demographic characteristics; practice characteristics; preferred approach to case involvement; and beliefs regarding care of children with behavioral/ psychosocial concerns.

To explore variables associated with GP/FPs' preferred approach to case involvement, we performed a series of logistic regression analyses using the two most clearly differentiated forms of involvement for each condition type (primarily manage by yourself vs. primarily refer out). The same independent variables were used as listed above, with the addition of respondents' Comfort/Skill scores for each condition type.

## Results

### Sample

Of 801 questionnaires mailed, 567 were returned, and 427 completed. A total of 172 recipients were identified as ineligible to participate, including 150 who did not complete questionnaires and 22 who completed the survey form in error, as they reported seeing no children with any of the study conditions in a typical month. The remaining 405 questionnaires formed the study database, a response rate of 64.4% of eligible recipients. Table 1 presents characteristics of the respondent group. In comparison with the non-responders, more respondents were female (37% vs. 29%, respectively; χ^2 ^(1) = 4.2; P < 0.05); certified as specialists in Family Medicine (40% vs. 28%, respectively; χ^2 ^(1) = 9.9; P < 0.005); and in group, as compared to solo, practice (63% vs. 51%; χ^2 ^(1) = 9.3; P < 0.005).

**Table 1:** Characteristics of the respondent sample

	**N**	(%)*
**Gender**		
Female	149	(37)
**Type of practice**		
Solo	93	(23)
Group	237	(59)
Walk-in	31	(8)
Other	44	(11)
**Location of practice**		
Urban	263	(65)
Rural	134	(33)
Other	7	(2)
**Time spent in patient care**		
40 hrs/wk or less	209	(52)
> 40 hrs/wk	191	(48)
**Recency of graduation**		
Prior to 1978	139	(34)
1978 to 1988	142	(35)
Since 1989	124	(31)
**Place of graduation**		
Canadian University	327	(81)
**Specialty certificate from the College of Family Practitioners of Canada**	166	(41)
**Participated in CME for psychosocial problems in children in past 5 years**	171	(42)
**Children seen per month: behaviour problems**		
None	28	(6.9)
1 to 4	324	(80.4)
5 to 9	46	(11.4)
>9	5	(1.2)
**Children seen per month: social-emotional difficulties**		
None	25	(6.2)
1 to 4	302	(75.0)
5 to 9	56	(13.9)
>9	12	(3.0)
**Children seen per month: ADHD/possible ADHD**		
None	81	(20.1)
1 to 4	303	(75.2)
5 to 9	18	(4.5)
>9	1	(0.2)
**Children seen per month: mood disorders/possible mood disorders**		
None	28	(6.9)
1 to 4	315	(78.2)
5 to 9	47	(11.7)
>9	13	(3.2)

Note: number of respondents varies slightly in tables of results because respondents did not always answer all questions fully

* Percentages rounded to nearest whole number

### Volume of patients seen with each condition

Between 75 and 80% of respondents reported seeing 1 to 4 children newly presenting with behavior problems or social-emotional difficulties, or in whom they would consider or make the diagnosis of ADHD or mood disorders, each month. Between 11% and 14% reported seeing 5 to 9 such cases for all conditions except ADHD, for which the proportion was 4.5%. Conversely, 20% of GP/FPs reported seeing no children in whom they would consider or diagnose ADHD, as compared with other condition types, for which the proportion varied from 6.2% to 6.9%.

### Preferences for case involvement

Respondents frequently reported combining personal involvement with referral. GP/FPs most commonly commence evaluation and management themselves, and then refer the patient for consultation (Table 2). We contrasted the frequency of GP/FPs who primarily evaluate and manage these cases themselves without referral, with the frequency of physicians who primarily refer out for evaluation and management or who refer out for evaluation and then take over management, across condition types. For children with behavior problems or ADHD, more GP/FPs referred cases than handled them by themselves (χ^2 ^(1) = 9.0; P < 0.005; and χ^2 ^(1) = 103.9; P < 0.001, respectively), whereas no differences in case involvement were found for children with social-emotional difficulties or mood disorders.

**Table 2:** GP/FPs' preferred approaches to case involvement*

	**Clinical Condition Type**			
	**Behavioral Problems N (%)**	**Social-Emotional Difficulties N (%)**	**ADHD N (%)**	**Mood Disorders N (%)**

Evaluate and manage the problem yourself	44 (11.1)	50 (12.7)	21 (5.3)	74 (18.8)
Evaluate and manage yourself, then refer for consultation	150 (38.0)	156 (39.5)	103 (26.0)	171 (43.4)
Evaluate yourself and then refer out for management	119 (30.1)	115 (29.1)	106 (26.8)	72 (18.3)
Refer out for evaluation and management	52 (13.2)	52 (13.2)	75 (18.9)	29 (7.4)
Refer out for evaluation and then take over management	25 (6.3)	17 (4.3)	82 (20.7)	36 (9.1)
Other combination of options	5 (1.3)	5 (1.3)	9 (2.3)	12 (3.0)

*Number (percentage) of GP/FPs that preferred each approach to involvement with each clinical condition type.

### Self-reported comfort/skill

Comfort/Skill scores by clinical condition type clustered closely around the midpoint of the 1 to 7 scale: behavior problems (mean ± s.d) 3.6 ± 1.1; social-emotional difficulties 3.8 ± 1.1; ADHD 3.9 ± 1.3; and mood disorders 4.4 ± 1.3. There was a statistically and clinically significant difference between Comfort/Skill with behavior problems and mood disorders (F [3,1155] = 84.0, P < .0001; effect size = 0.67), with respondents more positive about their ability to deal with mood disorders than behavior problems. This finding is supported and extended by the data on the frequency of physicians reporting "Low" vs. "High" Comfort/Skill (Table 3). High raters outnumbered Low raters by about 2:1 for all clinical activities related to mood disorders, whereas for most aspects of dealing with behavior problems, this pattern was reversed.

**Table 3:** Number (%) of GP/FPs reporting Low and High ratings of comfort, skill and effectiveness with each clinical conditions *

		**Behavior Problems N (%)**	**Social-emotional difficulties N (%)**	**ADHD N (%)**	**Mood Disorders N (%)**
Comfort with diagnosis/ evaluation	Low	147 (50.3)	111 (39.1)	136 (47.6)	86 (27)
	High	145 (49.7)	173 (60.9)	150 (52.4)	232 (73)
Skill in diagnosis/ evaluation	Low	183 (61.6)	152 (53)	144 (51.8)	96 (31.1)
	High	114 (38.4)	135 (47)	134 (48.2)	213 (68.9)
Comfort with management	Low	213 (70.5)	172 (59.1)	156 (51.1)	115 (35.5)
	High	89 (29.5)	119 (40.9)	149 (48.9)	209 (64.5)
Effectiveness in management	Low	223 (76.4)	192 (67.4)	148 (50.7)	104 (34.7)
	High	69 (23.6)	93 (32.6)	144 (49.3)	196 (65.3)

*Percentages are proportion of respondents with High or Low ratings, not of the entire sample.

### Beliefs about psychosocial problems in children

On the 1 to 7 scales, respondents indicated an overall tendency to disagree with the following belief statements: "These problems/conditions are usually mild and transient, so specific intervention or treatment is not usually required"; and "The role of primary care physicians should be very limited with these kinds of problems/conditions", with mean (s.d) ratings of 2.2 (± 1.2) and 2.4 (± 1.2), respectively. Conversely, there was a tendency to agree with the following statements: "These problems/conditions are usually related to stresses in the family which are hard to manage" and "Diagnosis/evaluation of these problems is often subjective and difficult", with ratings of 4.9 (± 1.3) and 4.4 (± 1.5), respectively (Table 4)

**Table 4:** Number (%) of GP/FPs reporting different levels of agreement with belief statements about primary care management of children and youth with psychosocial problems

Belief*	**Level of Agreement**		
	
	**Strongly Disagree **(Rating 1–2) **N (%)**	**Neutral Range **(Rating 3–5) **N (%)**	**Strongly Agree **(Rating 6–7) **N (%)**
**1**	270 (68.2)	121 (30.5)	5 (1.3)
**2**	19 (4.8)	245 (61.7)	133 (33.5)
**3**	252 (63.5)	73 (34.5)	8 (2.0)
**4**	61 (15.4)	232 (58.8)	102 (25.8)

* Belief statements:

1. These problems/conditions are usually mild and transient, so specific intervention or treatment is not usually required.

2. These problems/conditions are usually related to stresses in the family which are hard to manage.

3. The role of primary care physician should be very limited with these kinds of problems/ conditions.

4. Diagnosis/evaluation of these problems is often subjective and difficult.

### Factors associated with self-reported comfort/skill

In the multivariate models, two variables were related to higher Comfort/Skill across all condition types: having participated in CME to do with children's psychosocial problems in the past 5 years; and disagreement with the belief that evaluation of these conditions in children is subjective and difficult. Three other variables were related to Comfort/Skill across most of the condition types: seeing more children per month with that condition; disagreement with the belief that the role of the GP/FP in addressing these problems should be limited; and disagreement with the belief that these problems are related to stresses in the family that are hard to manage (see Table 4). No individual factor independently explained more than 7% of the variance in Comfort/Skill scores, but together each set of variables explained up to 20% of the variance in Comfort/Skill scores for each condition.

**Table 5:** Factors associated with higher self-rated comfort/skill

	** β **	**P**	**Unique Variance Explained (%)**	**Overall Variance Explained (%)**
**Behavioral Problems**				**19**
Participated in CME~	0.16	0.001	2.5	
See more than 5 children per month with behavior problems	0.14	0.003	1.9	
Belief: problems are mild & transient, specific intervention not required	0.12	0.01	1.4	
Belief: these problems are related to stresses in the family that are hard to manage	-0.13	0.007	1.5	
Belief: role of GP should be very limited	-0.19	0.000	2.9	
Belief: evaluation of these problems is often subjective and difficult	-0.22	0.000	4.2	
**Social-emotional Difficulties**				**19**
Participated in CME~	0.20	0.000	4.1	
Belief: role of GP should be very limited	-0.24	0.000	5.2	
Belief: evaluation of these problems is often subjective and difficult	-0.26	0.000	6.7	
**ADHD**°				**20**
Male gender	0.20	0.000	3.9	
Participated in CME~	0.21	0.000	4.4	
See more than 5 children per month with ADHD°	0.21	0.000	4.3	
Certified as specialist in Family Medicine	0.14	0.002	2.0	
Belief: these problems are related to stresses in the family that are hard to manage	-0.14	0.003	1.8	
Belief: evaluation of these problems is often subjective and difficult	-0.13	0.005	1.6	
**Mood Disorders**				**19**
Participated in CME~	0.22	0.000	4.5	
See more than 5 children per month with Mood Disorders	0.17	0.000	3.4	
Spend more than 40 hours/wk in patient care	0.10	0.04	0.9	
Belief: these problems are related to stresses in the family that are hard to manage	-0.09	0.05	0.8	
Belief: role of GP should be very limited	-0.18	0.000	2.9	
Belief: evaluation of these problems is often subjective and difficult	-0.16	0.001	2.3	

~CME: continuing medical education activities

°ADHD: attention-deficit/hyperactivity disorder

### Factors associated with preferred approach to case involvement

In our logistic regression analyses, only Comfort/Skill was related to preference for case involvement. Higher ratings of Comfort/Skill were strongly associated with lower likelihood of GP/FPs referring these cases out, with odds ratios (95% confidence intervals) as follows: for behavior problems, 0.16 (0.07 – 0.35; n = 114); social-emotional difficulties, 0.21 (0.10 – 0.44; n = 108); ADHD 0.26 (0.14 – 0.50; n = 170); and mood disorders, 0.21 (0.11 – 0.40; n = 128). Higher Comfort/Skill was therefore predictive of a tendency for GP/FPs to deal with these cases themselves. The proportion of variance (Nagelkerke R^2^) in GP/FPs' likelihood of referring out (rather than handling by him/herself) explained by Comfort/Skill was 61% for behavior problems, 53% for social-emotional difficulties, 34% for ADHD, and 60% for mood disorders.

## Discussion

GP/FPs in the Canadian province of BC, who are the predominant providers of primary health care to children, report a fairly consistent exposure to common childhood behavioral and emotional concerns in their practices. They report an intermediate level of comfort and skill in dealing with these patients overall, with the highest level for mood disorders and lowest for behavior problems. Caring for these children frequently involves collaboration with consultants. Self-reported comfort/skill was an important predictor of a GP/FP's tendency to take primary responsibility for a case, and self-reported comfort/skill was in turn related to previous educational exposure to this field, and beliefs about mental health problems in children. These findings have implications for physician education and primary care practice and organization of services.

Our results are consistent with other studies that have found an important role for physician self-efficacy in predicting physician practices [13,14], as would be predicted from social cognitive theory [15]. A previous study found pediatrician confidence in diagnosis and management of children and youth with depression to be associated with higher perceived responsibility for treating these cases, which is in turn predictive of the physician's prescribing medication and scheduling further appointments [10]. Although our results explain only a portion of the variation in physicians' comfort and skill, the consistent predictions from CME related to children's behavioral and emotional problems, and physicians' beliefs about the care of these problems, are notable. Participation in educational programs has been found to increase physicians' sense of professional efficacy in other studies [16], underlining the potentially important role for appropriate CME. Our finding that participation in CME and physician beliefs are both important factors in self-efficacy, raises the intriguing possibility that some of the benefits of participation in CME may be mediated through effects on attitudes and beliefs over-and-above simple knowledge acquisition. It has, in fact, been suggested that focusing on attitudes and beliefs may be a legitimate and important way to enhance skills and confidence among GP/FPs in relation to mental health problems [17].

We would point out that our results should not be interpreted as showing a direct benefit of physician CME on physician practice with children with behavioural and emotional problems, or on patient outcomes. The likelihood of physician CME resulting in changes in physician behavior and patient outcomes depends in large part on the type of CME activity undertaken, with certain types of CME being relatively ineffective and others being quite effective [18-20]. Our survey did not enquire about the types of CME activities in which physicians had participated. In relation to educational effects more broadly, previous studies from the USA have reported mixed results for effects of specialized training in psychosocial issues on the practice of primary care physicians with children with these problems. In one study, no effect of such training on treatment decisions was found [21], while in another study, more intensive levels of advanced training did result in better identification and management practices [22].

Our study raises questions about why GP/FPs feel more comfortable and confident in dealing with children with mood disorders, and less so those with behavior problems, and why they tend to refer children with behavior problems and ADHD more frequently than dealing with these cases themselves. Children's behavior problems may pose challenges because clear diagnostic criteria and management algorithms are lacking for this diffuse and heterogeneous group of clinical scenarios. An increased emphasis during physician training on understanding child development, and on learning practical strategies (such as contingent reinforcement) to deal with aberrant behavior, may enable GP/FPs to feel more confident with these problems. ADHD, on the other hand, continues to be a source of concern for many GP/FPs, in spite of accepted diagnostic criteria, evidence-based practice guidelines, and effective treatments.[23] Previous research from Australia identified diagnostic issues and complexities, time intensiveness, and insufficient education and training as contributing to GP/FP's reluctance to take primary responsibility for children with ADHD[8], while a US study of primary care providers found that higher severity of child psychosocial problems, and poorer family functioning, predicted referral to specialized mental health services [21].

The higher reported level of involvement and comfort/skill in dealing with mood disorders may reflect primarily work with adolescents, in which GP/FPs utilize strategies that are successful with adult patients, and drawing on the fairly extensive exposure to psychosocial medicine in family practice training[24]. We cannot address this possibility directly from data obtained from respondents in our survey, but we note that over 2/3 of pediatric cases of depression seen in a recent study of US pediatricians, did indeed involve youth 13 – 18 years of age [10]. We note, however, that the diagnostic and treatment approaches used for adults may not be appropriate for younger children, especially in light of concerns about the effectiveness and safety of specific serotonin reuptake inhibitor (SSRI) medications that have arisen since the time of our survey [25]. An apparent trend for more respondents to report high rather than low levels of comfort/skill for mood disorders compared with social-emotional difficulties, may be attributable in part to the terminology used in the survey. Although behavioral and social-emotional problems that would be considered subthreshold for specific diagnoses occur relatively frequently in primary care settings [26,27], it is possible that these non-specific and somewhat ambiguous terms may evoke uncertainty in the mind of clinicians trained in diagnostic and management approaches aimed at clearly delineated entities. Physician's responses in this survey might therefore reflect either uncertainty about how to cope with such problems in children, or perhaps about what was being referred to in the survey.

Strengths of our study include its careful sampling base of GP/FPs from across the province of BC, and its breadth and depth of scope. This is one of very few studies to examine how GP/FPs as a group, deal with children presenting with a range of behavioral and emotional problems, while at the same time exploring factors that underlie some of the physicians' practice patterns, preferences and perceptions. Certain limitations of this work also need to be acknowledged. Cross-sectional studies are limited in their ability to arrive at conclusions about causality. Hence we cannot be sure, for example, that attending CME increases comfort and skill levels. It is possible that physicians who attend CME on certain topics may be particularly interested and motivated in those areas. Nevertheless, our study highlights important associations, a number of which fit with causal expectations suggested by other studies and theory. We also cannot be sure that the views of our respondents reflect all GP/FPs' perceptions and practices, given our 64% response rate, although this was considerably higher than the 54% reported for physician postal surveys overall [28]. Finally, the extent to which our findings from a Canadian health care context would generalize to settings where patients' medical needs are not covered under a system of universal health insurance coverage, or where GP/FPs are less extensively involved in primary care, is unknown. However, our findings are consistent with those from other settings.[6-8,11] It is interesting to note that while primary care pediatricians in the USA report relatively low levels of confidence in their diagnostic and management skills for depression in children and youth most still become directly involved in some aspect of these patients' care [10], in a similar fashion to GP/FPs in BC. These observations underline the need for better support for primary care physicians in their role with these children, through increasing self-confidence and self-efficacy.

## Conclusion

It is encouraging to find that, in general, GP/FPs in BC report at least an intermediate level of comfort and skill in assessment and management. For the majority of patients, care involves the GP/FP and referral for specialist consultation. Given that specialists with skill and experience in dealing with psychosocial and mental health concerns are often in short supply, however, it would be desirable to enable GP/FPs to deal with more of these cases independently but confidently. To do so may require new educational strategies aimed at enhancing self-efficacy and capable of affecting attitudes and beliefs regarding mental health problems in children. In addition to these strategies, however, foundational changes in the way medical and support services are organized for care of children with psychosocial and mental health problems will also be required, given the barriers and challenges posed by financial and cultural factors, lack of access to specialized mental health services, and time and resource constraints within primary care [8,10,29].

## Competing interests

The author(s) declare that they have no competing interests.

## Authors' contributions

AM, CJ, AK and SF jointly conceived the study. AM, CJ, AK, SF and MP contributed to the study design. AM was responsible for overseeing day to day coordination of the study, anddrafted the manuscript. MP performed the statistical analysis. All authors contributed to interpreting the results, and read and approved the final manuscript.

## Pre-publication history

The pre-publication history for this paper can be accessed here:


